# Right Hepatic Artery Pseudoaneurysm Following Laparoscopic Cholecystectomy: A Rare Complication Treated With Coil Embolization

**DOI:** 10.7759/cureus.89125

**Published:** 2025-07-31

**Authors:** Vishal Babbar, Ronit Biswas, Aneesh Kumar, Sweta Kumari, Arvind Pratap, Pramod K Singh, Vivek Srivastava, Mumtaz A Ansari

**Affiliations:** 1 Department of General Surgery, Institute of Medical Sciences, Banaras Hindu University, Varanasi, IND; 2 Department of Radiodiagnosis, Institute of Medical Sciences, Banaras Hindu University, Varanasi, IND

**Keywords:** case report, coil embolization, hepatic artery injury, laparoscopic cholecystectomy, pseudoaneurysm, right hepatic artery

## Abstract

Hepatic artery pseudoaneurysm (HAPA) is an uncommon but potentially life-threatening vascular complication following laparoscopic cholecystectomy, often presenting days to weeks postoperatively. We describe the case of a 34-year-old female patient who presented 45 days following surgery with recurring hematemesis, melena, abdominal pain, and jaundice. Ultrasonography with colour Doppler suggested a vascular lesion near the porta hepatis, and triple-phase CT angiography confirmed a right hepatic artery (RHA) pseudoaneurysm leading to intrahepatic biliary radical dilatation (IHBRD). The patient underwent successful coil embolization, leading to full recovery without complications. HAPA should be considered in post-cholecystectomy patients who exhibit upper gastrointestinal (GI) hemorrhage or hemobilia. Early imaging diagnosis using Doppler ultrasound and CT angiography is imperative for timely intervention. Coil embolization provides a minimally invasive, effective alternative to open surgical repair, reducing associated morbidity and mortality. This case underscores the importance of prompt recognition and endovascular management of vascular complications following biliary surgery to prevent fatal outcomes.

## Introduction

Laparoscopic cholecystectomy is a commonly performed, minimally invasive procedure with a low complication rate. However, vascular injuries occur in approximately 0.8% of cases, with hepatic artery pseudoaneurysm (HAPA) being a rare but serious complication resulting from direct or thermal arterial injury [1,2]. An important surgical consideration is the variability of the right hepatic artery (RHA), which is particularly relevant during dissection in Calot’s triangle. In classical anatomy, the RHA arises from the proper hepatic artery. However, in approximately 15% to 25% of individuals, the RHA is “replaced,” most commonly originating from the superior mesenteric artery (SMA) rather than the proper hepatic artery [3,4]. In some cases, the RHA may be “accessory,” meaning an additional RHA is present alongside the normal one. These aberrant arteries frequently pass close to or through Calot’s triangle and are at increased risk of injury during laparoscopic procedures. Intraoperative misidentification or inadequate visualization of these variants can lead to arterial laceration, thrombosis, or pseudoaneurysm formation. Therefore, careful dissection and identification of the RHA and its branches are crucial, especially in patients with dense adhesions or altered anatomy. HAPA can result in a life-threatening hemorrhage if not promptly diagnosed and treated. This case presents an right HAPA having delayed gastrointestinal (GI) bleeding post surgery, emphasizing diagnostic challenges because of the often-overlooked nature of such injuries. It underscores the significance of postoperative vigilance, particularly in settings with limited awareness of vascular complications. Imaging modalities such as Doppler ultrasound and CT angiography are essential for diagnosis [5,6]. The Society for Vascular Surgery (SVS) 2020 guidelines endorse endovascular coil embolization as the preferred treatment for hepatic artery aneurysms (HAAs), citing its superior safety and efficacy compared to open repair. An endovascular-first approach is recommended in all anatomically feasible cases if arterial circulation to the liver can be maintained [7]. Early recognition and intervention are vital to minimize morbidity and mortality [2,6]. This case report follows the Surgical CAse REport (SCARE) 2023 checklist [8].

## Case presentation

A 34-year-old female patient with no known comorbidities presented to the surgical emergency department with a history of three episodes of hematemesis over the past month, including one episode on the day of presentation. The hematemesis was described as coffee-ground in appearance, with a projected volume of approximately 50-100 mL per episode. She also mentioned two episodes of melena in the last six days, intermittent epigastric abdominal pain, and jaundice (yellowish discoloration of the eyes, urine, and skin).

There was no history of fever, vomiting, abdominal distension, or altered bowel habits. The patient had undergone a laparoscopic cholecystectomy 45 days prior at another hospital. The immediate postoperative period was uneventful, and therefore, she was discharged on postoperative day (POD) 4 with a drain in situ. The drain was removed on POD 9, with minimal serous output. The first episode of upper GI bleeding occurred 20 days after the laparoscopic cholecystectomy, for which she was managed conservatively for three days at another hospital.

She had no significant medical or surgical history, except for two lower-segment cesarean sections (LSCS) performed five and three years ago, respectively. The patient was not on any regular medications before presentation. There was no family history of inheritable diseases or vascular abnormalities. No familial bleeding disorders or gastrointestinal conditions were documented.

On initial examination, the patient was alert and oriented, with stable vital signs: blood pressure was within normal limits, with a regular pulse rate, normal respiratory rate, and she was afebrile. She appeared pale and icteric, suggestive of anemia and jaundice. There were no signs of respiratory distress or hemodynamic instability.

Abdominal examination revealed four healed laparoscopic port site scars located in the epigastric, supraumbilical, right lumbar, and right subcostal regions. A transverse healed scar approximately 12 cm in length was noted in the suprapubic area, consistent with prior LSCS. The abdomen was soft with mild tenderness in the epigastric region. No guarding, rigidity, or palpable mass was present. On palpation, no signs of hepatomegaly or splenomegaly were seen; bowel sounds were present and normal. No peripheral stigmata of chronic liver disease, such as ascites, spider angiomata, or palmar erythema, were present. The cardiovascular and respiratory examinations were unremarkable.

The patient was kept nil per os (NPO) after being admitted to the hospital. Adequate intravenous fluids and proton pump inhibitors (PPIs) were started. Routine blood investigations were reviewed. The complete blood count (CBC) revealed anemia (hemoglobin: 7.67 g/dL (range: 13-17 g/dL)) and leucocytosis (15640/mm³ (range: 4000-10000/mm³)), suggestive of an ongoing inflammatory or stress response. The liver function tests (LFTs) revealed elevated total bilirubin (8.57 mg/dL (range: 0.3-1.2 mg/dL)), direct bilirubin (5.03 mg/dL (range: <0.2 mg/dL)), indirect bilirubin (3.54 mg/dL (range: 0.2-1.0 mg/dL)), alkaline phosphatase (973 IU/L (range: <240 IU/L)), and gamma-glutamyl transferase (GGT) (71 IU/L (range: 5-40 IU/L)), indicating cholestasis. Prothrombin time/international normalized ratio (PT/INR) revealed no abnormality, effectively ruling out coagulopathy. Renal function tests, as well as serum electrolytes, were also within normal limits. The patient’s hemoglobin level was optimized with transfusion of two units of packed red blood cells (PRBCs).

**Figure 1:**
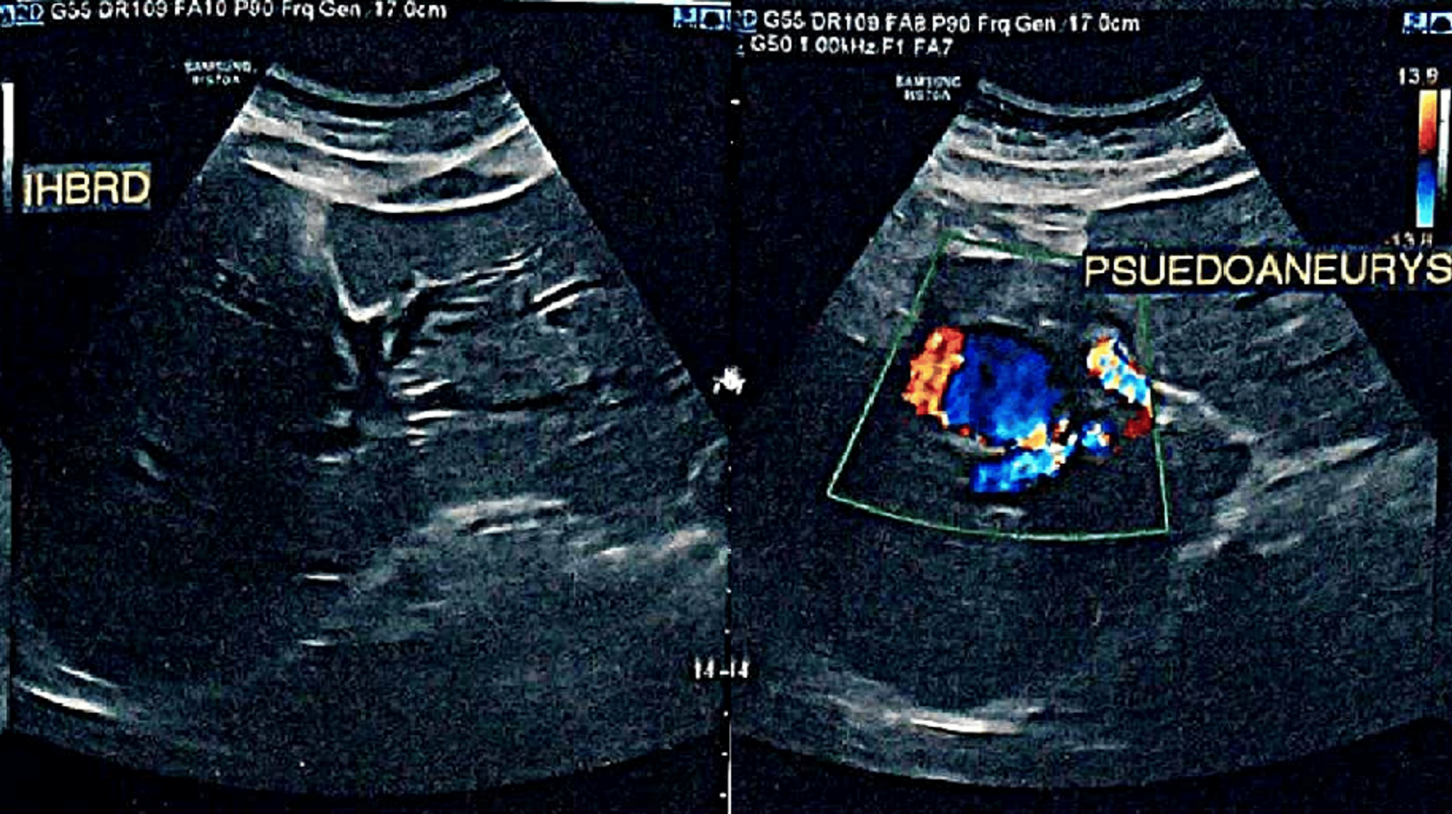
USG of the whole abdomen and color Doppler: moderate intrahepatic biliary radical dilatation (IHBRD) noted (central, bilobar); at the porta, a hypoechoic cystic lesion noted with the yin-yang sign on color Doppler, with the possibility of a pseudoaneurysm arising from the hepatic artery.

**Figure 2:**
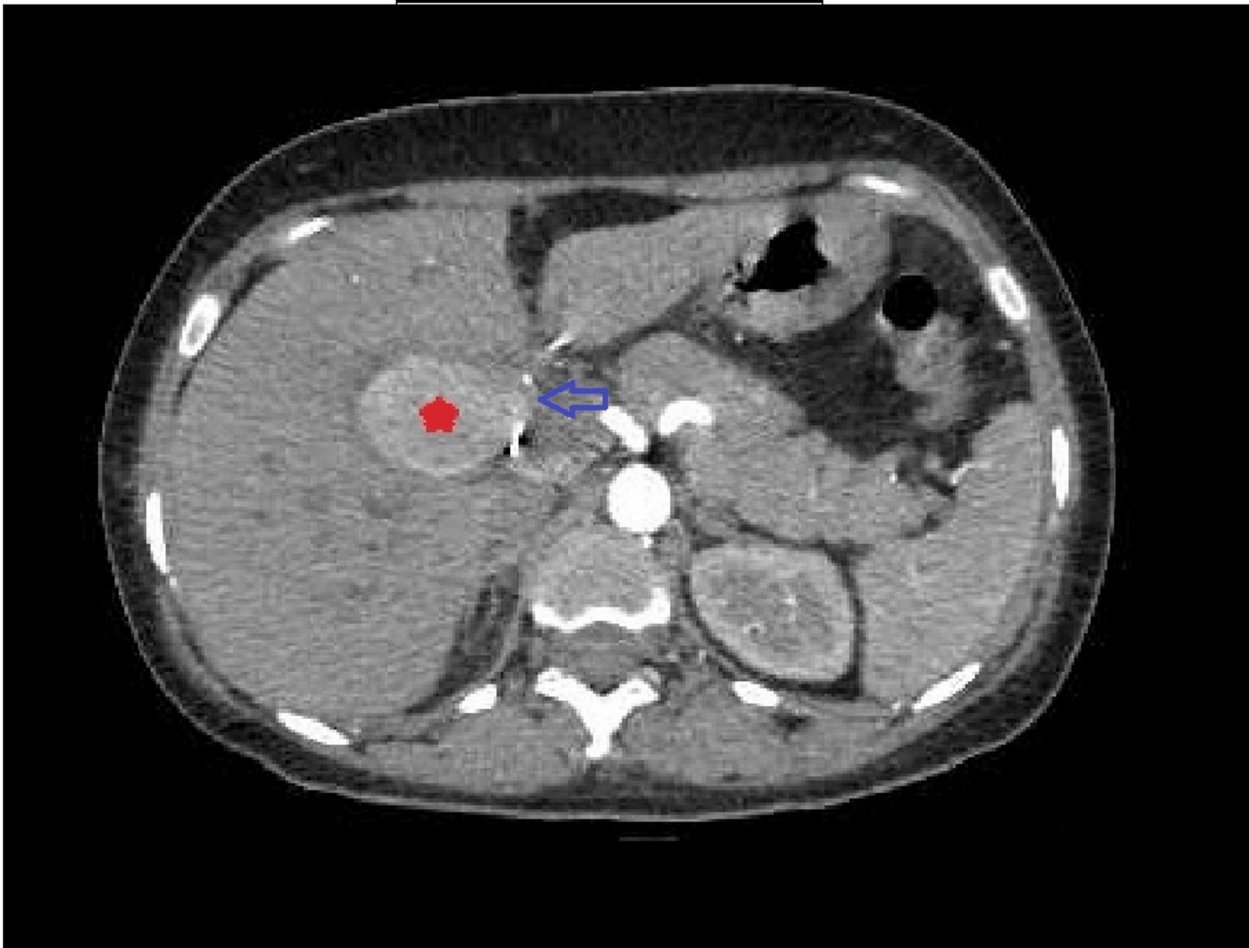
Triple-phase CT abdomen with angiography (arterial phase) demonstrating a well-defined right hepatic artery pseudoaneurysm (marked with a red star), 4 x 3 cm in size, neck diameter approx. 4 mm (marked with a blue arrow), 1.9 cm distal to the origin from the common hepatic artery, showing intense enhancement in the arterial phase with persistent enhancement in the venous phase. Bilobar intrahepatic biliary radical dilatation (IHBRD) (left > right) due to compression by right hepatic artery pseudoaneurysm

**Figure 3:**
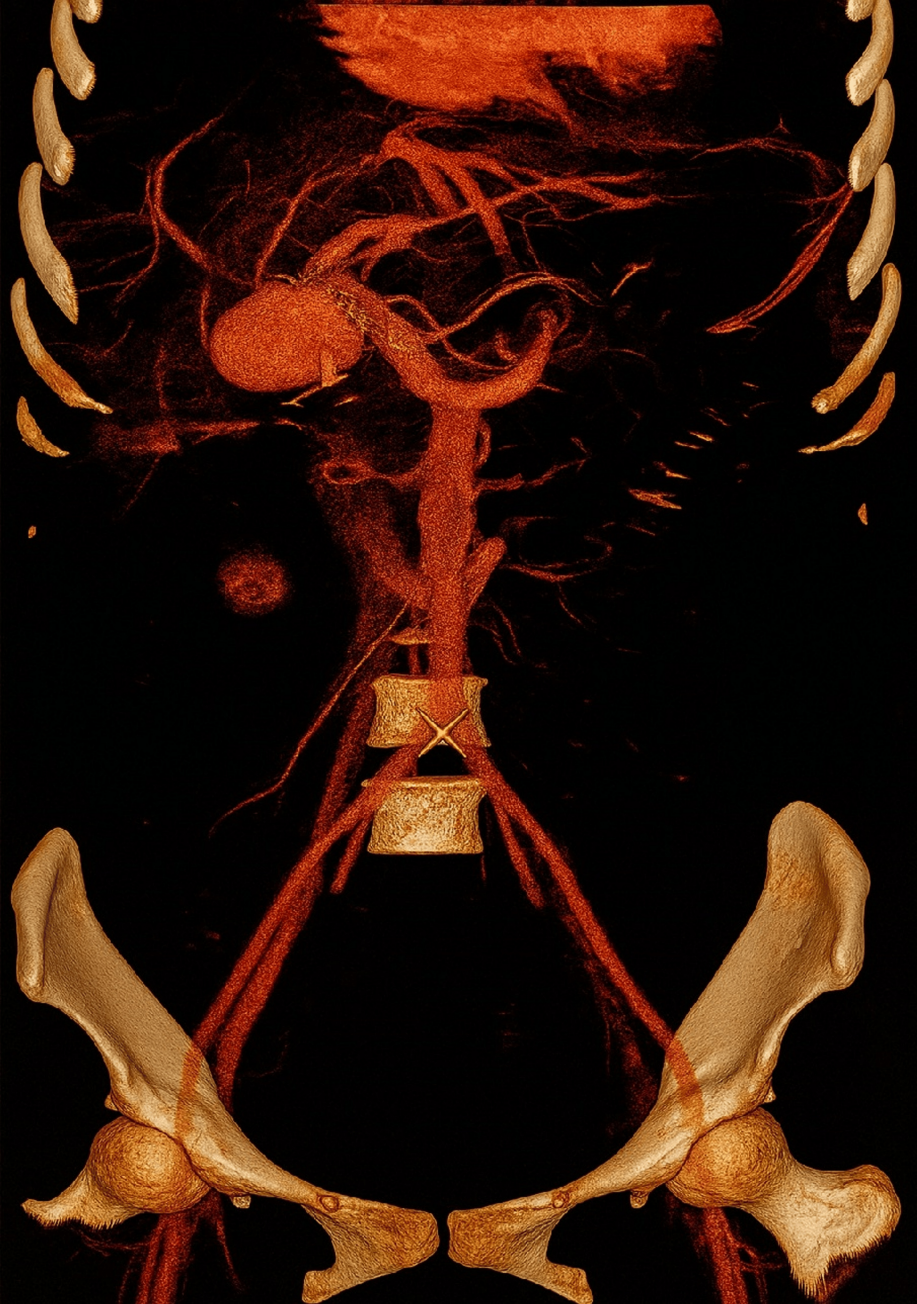
Three-dimensional volume rendering technique (3D-VRT) reconstructed contrast-enhanced CT (CECT) shows a pseudoaneurysm in the right hepatic artery territory.

An ultrasound with color Doppler (Figure 1) was performed, which was suspicious for HAPA. A subsequent triple-phase CT angiography (Figure 2 and Figure 3) was done, which confirmed the diagnosis of right HAPA.

The patient, along with her family, was counselled about the diagnosis, the need for endovascular intervention, and the potential risks involved. Informed written consent was obtained prior to the procedure.

**Figure 4:**
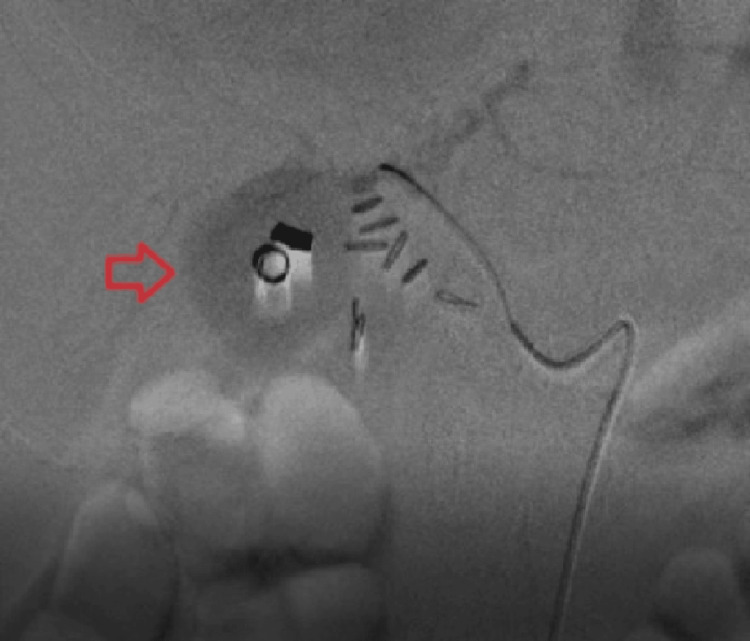
Pre-coiling digital subtraction angiogram showing contrast-filled outpouching (blush) in the right hepatic artery territory, marked with a red arrow, confirming a pseudoaneurysm.

**Figure 5:**
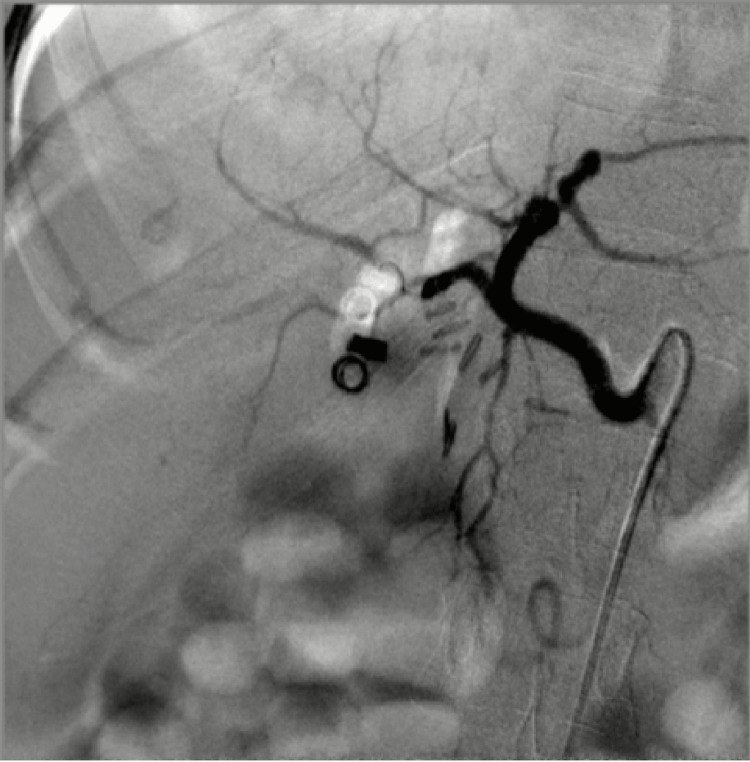
Post-coiling angiogram showing complete occlusion of the right hepatic artery pseudoaneurysm (HAPA), with no residual contrast blush, confirming effective coil embolization

The patient subsequently underwent endovascular coil embolization of the right HAPA (Figure 4 and Figure 5) using pushable platinum coils (NESTER® Cook® Medical, MWCE-18-3-2-NESTER-01, Cook Incorporated, Bloomington, IN). The procedure was performed by a senior interventional radiologist and resulted in complete exclusion of the pseudoaneurysm with resolution of bleeding and prevention of rupture.

The post-embolization course was uneventful. The patient was observed for seven days following the procedure and discharged on the eighth post-embolization day.

Follow-up evaluations at two weeks and one month in the outpatient clinic revealed that the patient remained clinically stable, having no recurrence of hematemesis or jaundice. Liver function tests demonstrated gradual normalization. At one month post discharge, the patient was asymptomatic and had resumed her routine activities.

## Discussion

Right HAPA is a rare but potentially life-threatening vascular complication that might develop after hepatobiliary surgery, including laparoscopic cholecystectomy. In the current case, the patient had an upper GI bleed along with evidence of obstructive jaundice secondary to hemobilia and compression of bile ducts, an uncommon presentation that significantly complicates timely diagnosis.

Pathophysiologically, an intraoperative arterial injury in the RHA led to a pseudoaneurysm that intermittently bled into the biliary tree (hemobilia). The extravasated blood traveled through the common bile duct into the duodenum, causing melena and even coffee-ground hematemesis upon reflux and vomiting. Within the biliary system, the accumulation of blood clots caused cholestasis and IHBRD, manifesting as obstructive jaundice on presentation. This sequence of bleeding (hemobilia) into the biliary tract leading to GI hemorrhage and jaundice corresponds to Quincke’s triad, although the full triad is not always evident. In fact, fewer than one-third of hemobilia cases present with the classic Quincke’s triad of abdominal pain, jaundice, and GI bleeding [6].

In our patient, the triad was incomplete (significant bleeding and pain were present, while jaundice appeared later), which contributed to the diagnostic difficulty. Notably, there were no vascular abnormalities noted on any pre-cholecystectomy imaging; the routine preoperative ultrasound had only confirmed multiple calculi in the gallbladder lumen, the largest measuring ~16.7 mm in size, indicating that the pseudoaneurysm developed de novo as an iatrogenic complication of the surgery.

Pseudoaneurysm formation after cholecystectomy is unusual, with a reported incidence ranging from about 0.06% to 0.6% [6]. Among these rare occurrences, the RHA is by far the most commonly involved vessel; literature reviews suggest it accounts for approximately 85% to 90% of post-cholecystectomy pseudoaneurysms [6]. Pseudoaneurysm of the common hepatic artery is much less frequent, documented only in a small minority of cases [6]. Pseudoaneurysms are typically iatrogenic, arising from arterial wall damage during gallbladder dissection. They are primarily due to mechanical trauma (such as inadvertent laceration, overly aggressive clipping, or traction injury) to the artery, with thermal injury as a less common mechanism [1,2]. During laparoscopic dissection in Calot’s triangle, inappropriate application of energy can cause arterial injury; however, thermal damage often results in immediate cauterization of the vessel, so a pseudoaneurysm is more likely when there is a partial tear or puncture to the artery that allows it to continue bleeding subacutely [6].

These injuries usually have a delayed presentation: the pseudoaneurysm often enlarges silently, and patients present days to weeks post surgery with nonspecific symptoms such as GI bleeding (hematemesis/melena), abdominal pain, or rarely jaundice [2,6]. In our case, the first bleed occurred around postoperative day 20. The intermittent nature of the hemorrhage (with periods of quiescence between bleeds) led to an initial conservative management, which delayed definitive diagnosis. This highlights the diagnostic challenge, as HAPAs are not routinely screened for unless there is high clinical suspicion [2,5]. Clinicians may first attribute postoperative upper GI bleeding to more common etiologies (such as peptic ulcer disease, stress-related mucosal erosion, or variceal hemorrhage) rather than an iatrogenic vascular injury. Indeed, if endoscopy is performed during an interval when the pseudoaneurysm is not actively bleeding, it may fail to reveal a source, leading to a false sense of resolution. Such factors contributed to the delay in our patient’s diagnosis. This underscores the importance of maintaining a high index of suspicion for vascular complications in any patient with unexplained GI bleeding after hepatobiliary surgery [2].

In this patient, early use of noninvasive vascular imaging was pivotal once HAPA was suspected. Doppler ultrasonography of the upper abdomen detected an abnormal arterial flow signal and a pseudoaneurysmal sac near the porta hepatis, which raised the suspicion of a vascular lesion. This was promptly confirmed by contrast-enhanced triple-phase CT angiography, which definitively demonstrated the right HAPA with active contrast pooling and adjacent intrahepatic biliary dilatation. CT angiography is the diagnostic gold standard and is invaluable for pre-intervention planning, as it delineates the arterial anatomy [6]. Angiography not only localizes the pseudoaneurysm but also guides the therapeutic approach. In our patient, no evidence of alternative bleeding sources (e.g., peptic ulcer) was found, and the imaging findings directly pinpointed the pseudoaneurysm as the cause of the hemorrhage and biliary obstruction. 

HAPAs carry a high risk of rupture and exsanguinating hemorrhage if left untreated; reported mortality rates reach up to ~50% in cases of rupture [2]. Therefore, timely diagnosis and intervention are critical for a favorable outcome. The preferred treatment for HAPA is an endovascular embolization approach, in line with current best practices. Available evidence indicates that patients managed with transarterial coil embolization have high success rates and lower morbidity compared to those undergoing open surgical repair [2,6]. The Society for Vascular Surgery clinical practice guidelines recommend an endovascular-first strategy for visceral aneurysms like HAPA, provided that hepatic perfusion can be preserved [7].

In this case, once the pseudoaneurysm was identified, the patient was promptly taken for angiographic embolization. Under fluoroscopic guidance, a microcatheter was selectively advanced into the RHA branch feeding the pseudoaneurysm, and the lesion was successfully occluded using pushable platinum coils (0.018-inch Nester® coils, Cook Medical). The coils were packed until there was complete stasis of flow into the pseudoaneurysm; a post-coiling angiogram confirmed complete obliteration of the pseudoaneurysm with no residual contrast blush. In our experience, this coil embolization technique achieved immediate hemostasis and exclusion of the pseudoaneurysm while avoiding the need for open surgery. The patient’s hemorrhage stopped immediately after embolization; there were no further episodes of hematemesis or melena, and her obstructive jaundice gradually resolved as the hemobilia ceased. She tolerated the procedure well, with no complications such as hepatic infarction or coil migration. This outcome mirrors the excellent results reported in the literature: transarterial embolization of HAPAs has a success rate exceeding 90% in published case series [6].

This report also highlights the importance of considering vascular complications in the differential diagnosis of postoperative GI bleeding, especially when the bleeding presents in a delayed fashion (weeks after the surgery) rather than immediately in the postoperative period. Early deployment of appropriate imaging, first with noninvasive Doppler ultrasound, followed by confirmatory CT angiography, expedites diagnosis and enables timely intervention [6]. Interdisciplinary collaboration was crucial in our patient’s management. The seamless coordination between the surgical team and interventional radiologists allowed for real-time decision-making and intervention, which was instrumental in achieving a good outcome. Advanced endovascular procedures such as coil embolization can be safely and effectively performed even in resource-limited settings, as long as the requisite expertise and infrastructure are available [6].

In summary, an iatrogenic pseudoaneurysm of the hepatic artery should be suspected when a patient presents with unexplained upper GI bleeding (especially hematemesis or melena) and possibly jaundice after a cholecystectomy. In our patient, the upper GI bleed was conclusively attributed to hemobilia from the right HAPA. Prompt imaging (USG and CT angiography) identified the source, and definitive treatment via coil embolization successfully stopped the hemorrhage and resolved the biliary obstruction. As similar cases in the literature have shown [1,2,6], vigilance for this rare complication is warranted. Rapid recognition and an endovascular therapeutic approach are the keys to minimizing morbidity and preventing potentially fatal outcomes [2,6,7].

## Conclusions

A high index of suspicion is essential for the timely diagnosis of vascular complications following laparoscopic cholecystectomy. When an unexplained GI hemorrhage occurs in the postoperative period, triple-phase CT angiography should be used promptly to detect rare but severe causes, including HAPA. Endovascular coil embolization is an effective and less invasive intervention that has good results, along with less morbidity than open surgical intervention. Close cooperation between surgery and intervention radiology teams is paramount in making the right diagnosis and optimal management. The rare complication described in this report can raise clinical awareness and may be used in the future to create updated guidelines or early alert protocols.
